# PPARs in Eye Biology and Disease

**DOI:** 10.1155/2008/518790

**Published:** 2008-05-26

**Authors:** Suofu Qin, Roy S. Chuck

**Affiliations:** ^1^Retinal Disease Research, Department of Biological Sciences, Allergan Inc., RD3-2D, 2525 Dupont Drive, Irvine, CA 92612-1599, USA; ^2^Wilmer Eye Institute, Johns Hopkins University, Baltimore, MD 21287-9278, USA

Welcome to this special issue of PPAR research dedicated to “PPARs in Eye Biology and Disease.” PPARs are well known to regulate the expression of genes involved in lipid and glucose metabolism. Very recently, these transcription factors have been demonstrated to modulate proliferative, inflammatory, and oxidative stress responses, including those that happen in the eye. We have collected a comprehensive group of review papers that are focused on discussing the relationships of PPARs with choroidal neovascularization, inflammation, and redox balance, as well as perspective therapeutic potentials of PPAR modulators in eye diseases.

Angiogenesis is an important element of normal development and neovascularization which occurs normally in wound healing. However, neoangiogenesis is unfortunately also associated with various pathological ocular conditions including corneal neovascularization secondary to graft rejection and traumatic, chemical, and infectious insults; diabetic complications in both the anterior and posterior segments; retinoproliferative disease secondary to vaso-occlusive events; as well as choroidal neovascularization associated with trauma, high myopia, genetic disease, and age-related macular degeneration (AMD). Of these, AMD is currently the leading cause of blindness in the developed world. As such, much effort and expense is and has been invested in understanding and seeking cures for this devastating condition. Although there is little direct evidence linking PPAR action to AMD, there is a growing body of literature demonstrating that PPARs may be involved in various chemical pathways associated with AMD. In this issue, three papers authored by respected experts in the field are presented which review what we now know about the relationship between the 3 PPAR isoforms, *α*, *β*, and *δ*, and ocular angiogenesis with emphasis on AMD. Bishop-Bailey has reviewed PPAR*β*/*δ*-mediated angiogenesis in the context of ocular disorders. Gehlbach et al. have briefly discussed the PPAR-*α* ligands as potential therapeutic agents for wet AMD. Chan et al. have comprehensively described PPARs with the development of AMD. There now appears to be ample data in the peer-reviewed literature to encourage further study of the link between PPARs and AMD, and investigate the therapeutic potential of PPARs. In addition, an authoritative fourth paper authored by Pershadsingh is also offered to address PPAR*γ* agonists as potential therapeutics for non-AMD proliferative retinopathies.

Inflammatory signaling participates in the development of different forms of eye diseases. Inflammatory injury happens under the conditions in which pathoangiogenic signaling is activated in acute inflammatory responses; chronic inflammation is triggered by oxidative stress in diabetic retinopathy and atrophic AMD. The majority of reports documented in the literature support an anti-inflammatory role of PPARs, in particular PPAR*γ*, by blocking the release of inflammatory mediators from activated immune cells in vitro and dampening inflammation in animal models. Minghetti et al. have explored the roles of PPAR*γ* in microglial cell functions and therapeutic potentials of PPAR*γ* ligands on ocular diseases such as AMD, diabetic retinopathy, autoimmune uveitis, and optic neuritis. Yanagi has evaluated the role of PPAR*γ* in the breakdown of blood-retinal barrier, providing strong evidence that targeting PPAR*γ* would be beneficial to diabetic retinopathy via maintaining the integrity of blood-retinal barrier. Phipps et al. have extensively reviewed the literature regarding the role of lymphocytes in thyroid eye disease-related inflammation, offering PPAR*γ* ligands as a therapeutic approach via inhibition of inflammatory signaling in activated lymphocytes and fibroblasts.

The potential regulation of redox balance by PPARs in the eye has been recently suggested and may constitute a new, exciting research field over the next few years. Phagocytosis of tips of rod outer segments' selectively upregulates expression of PPAR*γ* in retinal pigment epithelial (RPE) cells, suggesting that PPAR*γ* activation might deal with oxidative stress during RPE cell phagocytosis. Oxidative stress is a major risk factor causing RPE cell degeneration since RPE cells are exposed to high levels of free radicals due to phagocytosis of oxidized photoreceptor outer segments, intense light irradiation, and high oxygen consumption in the macular area. PPAR*γ* ligands protect a variety of cell types from oxidative stress injury in vitro, including retinal cells, though no in vivo data are available yet. Chang et al. have briefly reviewed the cytoprotective effects of an endogenous PPAR*γ* agonist, 15d-PGJ_2_, on oxidative stress-induced RPE cell death.

PPARs are emerging as potential targets for drugs that might be used in the treatment of ocular diseases in which PPAR activities play a key role in disease pathology. It is our hope that this special issue will serve as a seed stimulating broad interests to pursue therapeutic avenues of PPARs in eye diseases. The outcomes of such investigations will undoubtedly shed light on the roles of PPARs in eye diseases and possibly identify new roles of PPARs in the etiology of eye diseases.

*Suofu Qin* *Suofu Qin* *Roy S. Chuck* *Roy S. Chuck*

